# Draft genome sequence of *Fusarium coffeatum* Iraq-1 isolated from *Borago officinalis* plant

**DOI:** 10.1128/mra.01061-26

**Published:** 2026-10-19

**Authors:** Aymen Jasim Mahdi, Ola Hadi Jaafar, Adnan A. Lahuf

**Affiliations:** 1 Plant Protection Department, Agriculture College, University of Kerbala 125663, Kerbala, Iraq; Rochester Institute of Technology, Rochester, New York, USA

**Keywords:** *Fusarium coffeatum*, draft genome, seed rot, pathogenic isolate, Karbala-Iraq

## Abstract

We report the draft genome sequence of *Fusarium coffeatum* isolate Iraq-1 obtained from seed rot-affected *Borago officinalis* in Karbala, Iraq. The genome is 43.517 Mb with 9,369 contigs. This genome provides a resource for understanding the genetic basis of *F. coffeatum* from Iraq and across more global contexts.

## ANNOUNCEMENT

Seed and seedling diseases remain a persistent threat to crop productivity and plant health worldwide. Among the fungal pathogens involved, members of the *Fusarium incarnatum-equiseti* species complex (FIESC) are increasingly recognized for their broad host range (1, 2). *Fusarium coffeatum*, a recently delineated species within the FIESC, has so far been reported from only a limited number of countries, and whole-genome resources for this species remain scarce, limiting studies of its pathogenicity and host adaptation (3).

*Fusarium coffeatum* Iraq-1 was isolated in 2025 from seeds of borage (*Borago officinalis* L.) infected with seed rot disease. Samples were collected from agricultural fields in Karbala Province, Iraq (32.6160° N 44.0249° E ). Seeds were surface-sterilized with 1% sodium hypochlorite, rinsed thoroughly with sterile distilled water, and air-dried, and then fissured on potato dextrose agar (PDA) under 25 ± 2°C for fungal isolation. Directly after isolation, borage seeds were inoculated with emerging colonies to confirm pathogenicity and fulfill Koch’s postulates before genome sequencing was performed on a pure isolate (4).

Identification of the isolate to the genus level was based primarily on morphological features, such as colony morphology, conidial characteristics, and phialide structures. The molecular analysis confirmed the species identification by sequencing the internal transcribed spacer (ITS) region amplified with a pair of ITS1/ITS4 primers (5). BLAST analysis against the National Center for Biotechnology Information (NCBI) database revealed over 99% sequence identity between the Iraqi isolate and reference sequences (MT742819.1 and PP059082.1) of *F. coffeatum*.

Genomic DNA was extracted from a 7-day-old pure culture grown on PDA using DNeasy Plant Mini Kit (Qiagen, Hilden, Germany) according to the manufacturer’s instructions and prepared for whole-genome sequencing, as previously described (6), then sent to BGI (Hong Kong, China) for whole-genome sequencing. Sequencing libraries were prepared with the MGIEasy Universal DNA Library Prep Set (MGI) and sequenced using 150-bp paired-end reads on the Illumina HiSeq platform.

A total of 8,052,568 raw reads were obtained. Reads were processed with SOAPnuke version 2 for adapter trimming, low-quality base trimming, and removal of short reads (7). Default parameters were used. A total of 2,415,770,400 clean bases were retained after filtering with a Q20 value of >97.58% and a Q30 value of >91.98%.

We assembled filtered reads *de novo* utilizing SPAdes version 3.15.5 (8) and scaffolded them in Geneious Prime against the scaffold of a closely related species from the FIESC (GCF_003316985.1). Default parameters were applied. Contigs and scaffolds above 200 bp were retained. Total assembly yielded 9,369 contigs, with a genome size of 43,517,250 bp, N50 = 63,008 bp, and L50 = 113, guanine–cytosine (GC) content of 47.6%, and estimated sequencing depth (based on non-redundant data) of approximately 18.0. However, the draft genome of *F. coffeatum* isolated in this study was unannotated.

Genome completeness was determined by BUSCO version 5.4.4 using the ascomycota_odb10 data set (9) with a completion of 96%. Species assignment was confirmed by average nucleotide identity (ANI) analysis using the FastANI tool (Galaxy Version 1.3) between the assembled genome and reference FIESC genomes (GCF_003316985.1) (10, 11). This analysis supported the classification of the isolate as *F. coffeatum* species with an ANI value of 96%.

## Data Availability

The sequence of the internal transcribed spacer (ITS) region of F. coffeatum isolate Iraq-1 was assigned with an accession number PZ873180.1. The genome data generated in this study have been openly available from NCBI under BioProject accession PRJNA1469889. The BioSample with accession number SAMN60386775 is associated with isolate Iraq-1 sequenced in this study. The whole-genome shotgun (WGS) assembly for this isolate has been deposited in DDBJ/ENA/GenBank under the accession number JBYQTJ000000000. All raw sequencing reads were deposited into NCBI Sequence Read Archive (SRA) with the accession number of SRR38865018.
